# Supplementary material for: Patterns of Intron Gain and Loss in Fungi
Source: PLoS Biol. 2004 Nov 30;2(12):e422. doi: 10.1371/journal.pbio.0020422 (PMC532390; doi:10.1371/journal.pbio.0020422)
Supplement: Table S1 — Also available at http://genes.mit.edu/NielsenEtAl/. (4.3 MB ZIP). [file pbio.0020422.st001.zip › NielsenEtAl/html/1385.html]

AN4051.1.NCU07352.1.MG03446.1.FG07925.1


```
 CLUSTAL W (1.82) Multiple Sequence Alignments - Introns Inserted


Sequence 1: MG03446.1	323 aa
Sequence 2: FG07925.1	263 aa
Sequence 3: NCU07352.1	317 aa
Sequence 4: AN4051.1	286 aa
Alignment Length: 341 aa
Number Identitical Residues: 109 aa
Alignment Score (without introns) 5733


MG03446.1 	---------MASDR---------LRMHVERRDLYTNLEARIHYLHSFLDFSSR~DIEALV
NCU07352.1	---------MASSASSSSARTRAPMQHIDRKELYTSLEARIRYLHSFLDFGTD1DVEALS
FG07925.1 	----------MNSR---------PMKHIDREDLYTNLEARVQYLHSFLDFSSR1DIEALI
AN4051.1  	MENQPARMSGNTDKLAYFKLDGREVKKISRKALYTRLEARINYLKDFLDFNSS1DMEALA
          	 ....:  :  ..  :          ::.*. *** ****:.**:.****.:  *:*** 

MG03446.1 	SGAKYVRALIPAVVNIVYKKLLRYDITARAFTTRSTSFEGPMDECPDEDSPQIQHRKVFL
NCU07352.1	SGAKYIQALIPAVVNIVYKKLLQYDITARAFQTRSTAFEGSMDEVPDEESPQILHRKLFL
FG07925.1 	TGAKYVKALIPAVVNIVYKKLLQYDITARAFTTRSTSFEGPLDEVPDENSPQILHRKMFL
AN4051.1  	SGAKYIKTLIPAVVNLVYRKLLEYDITARSFHTKDTASEAPIEDFYNEDSPPIMRRKMFL
          	:****:::*******:**:***.******:* *:.*: *..:::  :*:** * :**:**

MG03446.1 	RAYLSKLCSDPSQMEFWEYLDKVG2MMHVGLGRAHPLHVEYVHLGVCLAFIQDIMSEAIL
NCU07352.1	RAYLKKLCSDPTQMEFWEYLDKVG~MMHTGLGRTHPLHIEYIHIGVTLGVIQDILNEAIL
FG07925.1 	RAYLMKLCSDPSKMEFWEYLDKVG2MMHVGLGRKHPLHIEYVHLGVCLGFIQDIMTEAIL
AN4051.1  	RWYLTKIVTDPMQMDFWRYLNKVG2LMHTAQERIHPLNIEYIHMGACLGYIQDLFTEALL
          	* ** *: :** :*:**.**:*** :**..  * ***::**:*:*. *. ***::.**:*

MG03446.1 	SHPRMAMQNKIALVKALGKVIWIQNDLMAKWQVRDGAEFHTDDGSEEEIVIEKEGYLHGK
NCU07352.1	SHPRLPMPRKIAFVKALGKVIWIQNDLFTRWYVHDGEEFTDGMDFG---VVEKEGFLHGK
FG07925.1 	SHPRLHIQRKTALVKALNKVIWIQNDLMAKWHVRDGSEFEVGDS---DIEIEREGYLHGK
AN4051.1  	SHPTLSIARKTAMIRAISKIIWIQNDLIARWRMRDGEEFADEMSEY--IIDDKEGYLGDK
          	*** : : .* *:::*:.*:*******:::* ::** **    .       ::**:* .*

MG03446.1 	KILDEDDDVPEAVSPASSTSSQHTLTPPRSLLSPLSPTTCMPRRSTGSEGAGVCPFSGRQ
NCU07352.1	KVLDETAEASSSSAPAAATGGSACPFSSVVE-------SKDWAAAAASASDSTCPFSG--
FG07925.1 	RIIGDNS------GSASDDEASDQLPPSR-----------IP-HPNGASAGGVCPFSG--
AN4051.1  	KILGDSS--------SSSRSSSRSMEDDR--------------VSIAPSVASACPFAD--
          	:::.:          ::   ..                      . ..   ..***:.  

MG03446.1 	ANDPSTPERRKPDIKIEVSEPVTFAGSMQSLSGVALSGRTESI
NCU07352.1	ASKAMEGLSIKDKKPVSAAK---NNDDARSDTTEKASRQETAI
FG07925.1 	MGAPSEE------------------------------------
AN4051.1  	LAQPITQTKIWAGK-----------------------------
          	   .
```
